# The First Colored Dental School

**Published:** 1887-03

**Authors:** 


THE FIRST COLORED DENTAL SCHOOL.—*The Meharry Medical Commencement.*—The eleventh annual commencement of the Meharry Medical Department of Central Tennessee College, and the first commencement of the School of Dentistry, were held at Masonic Theater, Nashville, Tennessee, February 21st. Every available seat in that large building was filled, while hundreds could not gain admission; and yet perfect order reigned in the audience, some of whom were white citizens of Nashville, who are becoming interested in this work. Among those seated on the stage were the Governor of Tennessee, State Superintendent of Public Instruction, Secretary of the State Board of Health, Mr. Robert Blackstock and Mr. Frank Meharry, of Illinois, Hon. R. P. Yancey, Hon. E. I. Golliday, Hon. Dr. Harwell, Col. A. S. Colyar, Speaker Ewing, of the State Senate, and many members of the General Assembly of Tennessee, and a number of leading citizens of Nashville.

The salutatory oration was delivered by Rev. W. A. Sinclair. His subject was "The March of Medical Science." He traced the history of medicine from the earliest ages down to the present time, in pleasing language and intelligent manner.

The valedictory, by J. W. Pickett, on "The Diseases of the Heart," was well delivered. In conclusion he referred very feelingly to their gratitude to those who have shown an interest in the elevation of the race. He said: "There are some hearts which are grateful unto death; let not a shade of doubt ever come to divert your interest from us; feel not that the Negroes are ungrateful for what has been done to help them."

The valedictory was followed by the charge to the medical department graduates, by G. W. Hubbard, M. D., Dean of the College. He gave a short history of the growth of the Meharry Medical College. At the time it was organized, the question of educating Negro physicians was a new and unsolved problem. He said: "Eleven years ago this work began in one small room, without money, apparatus, or experience, and with only a handful of students. We have now a handsome building with ample grounds and growing accommodations, from which we have sent out sixty-two graduates besides the present class. At the beginning of this term a school of dentistry was opened, which is the first and only colored dental school in the South · and for which we are very much in need of a suitable building."

The address to the class in dentistry was delivered by W. H. Morgan, M. D., D. D. S., dean of the Dental Department ot Vanderbilt University. Dr. Morgan gave an interesting history of dentistry, going back to the time of Herodotus, who spoke of it in connection with medicine in Egypt. The degree of M. D., was then conferred by President Braden on ten young men, who had finished their course in medicine, and three received the degree of D. D. S.

Governor Taylor was called for, and, in response, said he had not come there to make a speech, but to show these young men his deep interest in their welfare by his presence; that their race was preparing to fight the battle of life, and that they were the advance guard of the civilization of their people. "Lift your race into a higher plane," he said, "that we may go on together to a grand and glorious future."

His remarks were followed by short speeches from Superintendent Payne; Secretary of Board of Health J. B. Lindsley, M. D.; A. S. Colyar, editor of the Nashville *Union*, and Mr. McElwee, one of the colored members of the General Assembly.

Nine dental and fifty four medical students have been enrolled the past sesion.

It is a most encouraging sign of future success, when a Governor of a Southern state, the editor of one of the leading public journals, and a dean of a department of Vanderbilt University favor an occasion of this kind with their presence and words of encouragement, and the leading daily paper of the city devotes an entire page to the report of the exercises, as the Nashville *American* did of this on February 22d.

